# Prevalence and risk factors of Apical periodontitis in endodontically treated teeth: cross-sectional study in an Adult Moroccan subpopulation

**DOI:** 10.1186/s12903-021-01491-6

**Published:** 2021-03-17

**Authors:** Imane El Ouarti, Sanaa Chala, Majid Sakout, Faiza Abdallaoui

**Affiliations:** 1 Department of Endodontics and Restorative Dentistry, Faculty of Dental Medicine, Mohammed V University in Rabat, Rue Mohammed Jazouli, BP 6212 Madinat Al Irfane, Rabat, Morocco; 2 Military Teaching Hospital Mohammed V, Rabat, Morocco; 3 Laboratory of Biostatistics, Clinical and Epidemiological Research, Faculty of Medicine and Pharmacy, University Mohammed V in Rabat, Rabat, Morocco

**Keywords:** Apical periodontitis, Endodontic treatment, Risk factors

## Abstract

**Background:**

The present study aimed at investigating the prevalence of Apical periodontitis in a Moroccan Adult subpopulation with a non-surgical root canal treatment and to assess associated risk factors including endodontic treatment quality, periodontal health status, coronal restoration cavity design and quality.

**Methods:**

A total of 358 endodontically treated teeth were evaluated after more than 1-year period in a Moroccan subpopulation according to predetermined criteria. Studied parameters were assessed clinically and radiographically. The association between coronal restoration quality, cavity design, periodontal status, root canal filling quality, coronal restoration related features, presence or absence of the opposing dentition and the periapical status was determined. Data were analyzed using chi-square test, odds ratio and logistic regression.

**Results:**

The present study revealed that gingival health, coronal restoration with CL II cavity design, and root canal filling quality influenced periapical status of endodontically treated teeth. Multivariate analysis showed that this association was statistically significant for gingival inflammation (95% CI 1.08–3.91, OR 2.05, p = 0.02), inadequate coronal restoration (95% CI 1.16–4.04, OR 2.16, p = 0.01), inadequate root canal filling length and homogeneity (95% CI 1.24–3.01, OR 1.93, P = 0.004), (95% CI 1.41–4.44, OR 2.50, p = 0.002) respectively.

**Conclusions:**

The present study revealed that inadequate coronal restorations especially with large proximal margins (CL II cavity design) and gingival inflammation increased the risk of apical periodontitis in endodontically treated teeth. Prevalence of Apical periodontitis in the present study was 72.1%.

## Background

Apical periodontitis (AP) is an inflammatory disease of periapical tissues and develops as a host’s immune response to the presence of microorganisms and their irritants within the root canal system [1]. In most of the cases, AP is a direct consequence of dental caries [2], nevertheless several authors have reported that AP is more prevalent in endodontically treated teeth (ETT) [3–5].

The ultimate goal of endodontic treatment is to eliminate or at least reduce the microbial load within the root canal system through chemo mechanical debridement followed by root canal filling [6]. However, several studies showed that apical periodontitis is still raising despite technical developments of root canal treatment procedures [7, 8].

A Meta-analysis of previous cross-sectional studies, has shown a significant higher prevalence of AP in treated teeth with inadequate root canal treatment (OR = 4.65; 95% CI 2.75–7.84; p < 0.00001), and a significant higher prevalence of AP in ETT with poor coronal restoration (OR = 1.54; 95% CI 1.16–2.05, p = 0.003) [3, 9–14].

An updated systematic review of cross-sectional studies published between 2012 and 2020 [9], revealed a worrying increase in the worldwide prevalence of AP among endodontically treated teeth (from 35.9% to 41.3%). These results enhance the need for further investigations of risk factors of AP in ETT, especially because of the growing evidence of AP impact on impaired systemic health (eg, diabetes mellitus, cardiovascular diseases) [15, 16].

Epidemiological studies also revealed that apical periodontitis in ETT has been associated with periodontal health status [17, 18] and with tooth structure loss. In a prospective study assessing the effect of coronal tooth structure loss on the outcome of root canal treatment, Al Nuaimi et al. [19], concluded that less tooth structure increased likelihood of endodontic failure after one year.

Previous cross-sectional studies investigating AP prevalence and AP risk factors present a high heterogeneity (Clinical or radiographic data assessment, two-dimensional or three-dimensional imaging techniques used, risk factors considered separately or analyzed together), these discrepancies between investigation methodologies, make interpretation of the available results with many uncertainties. Hence, the need for more studies with consistent and well-defined design guidelines in the future to achieve a reliable comparison between the different findings [20].

Only a few Data on AP prevalence and risk factors for the disease development were published in Africa, most of the previous studies, were carried out in European, American and Asian countries [5, 13, 21]. Therefore, more attempts should be provided to identify a complete picture on AP epidemiology and specific treatments needs in African countries, especially because of the socio-economic outcomes of this Oral health disease and its undeniable impact on general health in the general population [22, 23].

The aim of the present study was to assess in a Moroccan Adult subpopulation both clinically and radiographically prevalence of AP and risk factors associated to AP in ETT including endodontic treatment quality, periodontal health status, coronal restoration cavity design and quality.

## Methods

### Study design

This cross-sectional study was carried out between April and December 2019 on patients attending the Department of Conservative dentistry and endodontics within the dental faculty hospital, Rabat (CCTD Rabat). The study was conducted in compliance with the ethical principles stated in the declaration of Helsinki and was approved by the Research Ethics Committee, Mohammed V University, Rabat (comité d’éthique pour la recherche biomédicale de Rabat [CERB]) under ID Number 08/18) and the data assessment followed the "strengthening the reporting of observational studies in epidemiology" [24].

### Patient selection

The Study sample size was calculated by using the following formula:

$$n=\frac{{Z}^{2}P(1-P)}{{e}^{2}}$$

The prevalence (P) of AP was estimated at (63.79%) [21]. The Z value was 1.96 for 95% confidence interval, and the precision level (d) was determined at 0.05. From the above formula, the minimum sample size required was 358 patients. From 360 patients enrolled in the study, only 148 patients satisfied eligible criteria to be included according to their medical history, periapical radiographs quality. 369 teeth were assessed, 11teeth were excluded for being endodontically treated less than 1 year.

An initial pilot study was undertaken on 68 patients and 80 endodontically treated teeth, the preliminary results showed significant differences with respect to association between Apical periodontitis, root canal treatment quality, coronal restoration quality and cavity design, but not for association with periodontal health status. The sample size was further extended to reach the calculated sample size and lead to powerful results.

Clinical data assessment was performed by one examiner, uncertain situations were discussed with two other observers. A single observer (IE) was calibrated before evaluating the radiographs. Intra-observer agreement for periapical status scores, root canal filling quality, coronal restoration quality was assessed by calculating Cohen’s Kappa coefficient (*k*) after re-examining a random sample of 30 of the periapical radiographs.

#### Inclusion criteria

- Adult subjects 18 years or older with no medical history who agreed to participate in the study.
- Teeth endodontically treated for more than 1 year.
- Periapical radiographs with no processing artefacts allowing visualization of the investing bone beyond the radiographic apex at 2 mm at least with good density and contrast.

#### Exclusion criteria

- Patients with medical conditions that may affect the healing process or the immune system conditions included: Diabetes, chemotherapy, Jaw bones radiotherapy, autoimmune diseases and patients taking any medication known to alter metabolism such as: immunosuppressive drugs, corticosteroids, Biphosphonates.
- Immature teeth
- Teeth that have undergone endodontic surgery and apical resection.
- Missing information or incomplete record.

#### Clinical and radiographic examination

Patients participating in the study and seeking routine dental care (no emergency care) were examined clinically and radiographically. All radiographs had been examined using an x-ray viewer under good illumination and 5× magnification, optimal conditions were adopted for the best possible radiographic contrast.

A clinical examination was carried out including periodontal probing, palpation of the adjacent mucosa and vertical percussion of the endodontically treated teeth. The coronal restorations were clinically assessed using explorary probe 6 (Dentsply Maillefer, Ballaigues, Switzerland).

### Data collection

For each subject, the following informations were recorded:

- Patient’s age and Sex.
- Tooth group: incisor, canine, Premolar or Molar.
- Existence of opposing tooth: opposing tooth present or absent.
- Date of root canal treatment completion.
- Type of coronal restoration of the endodontically treated teeth:
  - full coverage crown: restoration of the coronal part of the tooth which appeared to be a cast restoration or a porcelain crown.
  - coronal filling inserted during plastic phase: restoration of the coronal part of the tooth which appeared radiopaque on the radiographs.
- The coronal filling material was specified: a composite resin or amalgam restoration.
- cavity design: the cavities were matched according to cavity type (Black’s classification).
- Periodontal health status was classified as follows:

Presence of dental plaque, bleeding, calculus during probing, gingival recession, loss of periodontal attachment, pocket depth, bone loss in the alveolar crest or in the furcation area and tooth mobility were considered in the assessment of periodontal health. All the periodontal pockets deeper than 4 mm were considered pathologic accordingly with the criteria established by the American Academy of Periodontology (2015) [25].

- Healthy: no visible biofilm, no gingival bleeding on periodontal probing, physiologic tooth mobility, no probing depth recorded on buccal, lingual, mesial and distal surfaces.
- Periodontal disease: presence of gingival bleeding on periodontal probing, pocket depth ≥ 4 mm, bone loss visible on radiographs, pathologic tooth mobility due to insertion loss.

Periapical status was classified as follows:

- Healthy: No clinical symptoms, no rarefaction of bone visible on the radiograph, periodontal ligament of normal width or slightly widened.
- Apical periodontitis: clinical signs and symptoms of periapical inflammation, infection or both, widened periodontal ligament, presence of any discernible periapical radiolucency.

The periapical status of endodontically treated teeth was evaluated clinically and radiographically, and classified as healthy or diseased using a combination of the PAI Scoring system [26] and radiographic criteria used by De Moor et al. and Song et al. [27, 28]. The worst periapical status of all canals was taken to represent the periapical status in multi-rooted teeth.

Coronal restoration quality was assessed as follows:

- Adequate: any permanent restoration that appeared clinically and radiographically intact.
- Inadequate: any permanent restoration with detectable clinical and radiographic signs of open margins, overhangs or caries, or no restoration at all.

Coronal restoration was clinically and radiographically determined to have well-sealed margins, to restore tooth anatomy and to be without any recurrent caries. Modified and simplified Ryge’s Criteria were used [29] (Table1).

**Table 1:** Coronal restoration quality and root canal filling quality scored on endodontically treated teeth

Variables	Score
Clinical and radiographic quality of coronal restorations	0: Anatomical restoration with no open margins, no overhangs, and no recurrent decay (Adequate)
	1: Restoration with overhangs (Inadequate)
	2: Restoration with open margin (Inadequate)
	3: Restoration with unsatisfactory anatomic form (Inadequate)
	4: Restoration with recurrent decay (Inadequate)
	5: Fractured, detached, or lost restoration (Inadequate)
	6: tooth cusp or tooth wall fracture (Inadequate)
*Radiographic quality of root canal filling*	
Length of the root filling	0: root filling ending from 0 to 2 mm short of the radiographic apex (adequate)
	1: root filling ending more than 2 mm short of the radiographic apex, unfilled canals, detectable void between the intra-radicular post and the filling material (inadequate)
	2: filling extruded beyond the apex (inadequate)
Homogeneity of the root filling	0: root canal filling with good radiographic density, no detectable voids between filling material and root canal walls (adequate)
	1: root canal filling with low radiographic density, detectable voids (inadequate)
Conicity of root canal filling	0: continuously tapering funneled preparation from the canal orifice to the apex, and cross-sectional diameter narrower at every point apically (adequate)
	1: inconsistant taper from the coronal to the apical part of the filling, or root filling deviated from the original canal (inadequate)

The root canal filling quality was classified as follows:

- Adequate: when all root canals were obturated with dense fillings, no visible space between the material and the walls of the canal or within the body of the obturating material, the root canal filling ending within 0–2 mm short of the radiographic apex with a consistent taper from the orifice to the apex.
- Inadequate: when the root canal fillings were poorly condensed, the canal space visible laterally and apically with no consistent taper and when the root canal fillings were overfilled or ended 2 mm shorter than the radiographic apex or in the presence of unfilled canals.

In Multi-rooted teeth, the worst root canal filling quality was considered.

The root canal fillings were evaluated according to the European Society of Endodontology (ESE 2006) [30]. Root canal conicity was evaluated on periapical radiographs of endodontically treated teeth according to Shilder’s design objectives for root canal shaping and filling as a continuously tapering funneled preparation from the canal orifice to the apex [31].

A well performed endodontic treatment was assessed according to the previous recommendations as dense with radiopaque appearance of the filled canals and no voids seen between canal filling and canal walls, and the root canal filling ending at 0–2 mm from the radiographic apex.

Table 1 shows criteria used to assess the quality of coronal restorations and root canal fillings.

### Data analysis

Data analysis was performed by using the SPSS Software (Statistical Package for the Social Sciences, version 13.0, IBM, Chicago, IL), continuous variables were presented as means and Standards deviation, or as medians and interquartile range, as appropriate. Categorical variables were presented as numbers and percentages. Association between risk factors and AP was identified by a chi-square test or Fisher’s exact test, as appropriate. Odds ratio (OR) and 95% confidence interval (CI) were also calculated for each association. Significance level was set at p < 0.05.

The main outcome measure was the absence versus the presence of apical periodontitis. Apical periodontitis (AP) was the dependent variable, explanatory variables (covariates) were coronal restoration quality, cavity design, coronal filling type and material, root canal filling quality, periodontal health, tooth location, existing of opposing tooth, age and gender.

Univariate and multivariate logistic regressions were used to relate patient and treatment variables with the AP prevalence. Explanatory variables to be included in multivariate analysis were identified in univariate analysis.

## Results

The intra-observer score yielded a Kappa value of 0.95, 0.95, 0.85 for the evaluation of periapical status, root canal filling quality, coronal restoration quality respectively, which indicates a good intra-observer agreement.

Of the selected study population of 148 patients, 112 (75.7%) were female and 36 (24.3%) were male. The average patient age was 41 ± 11.7 years. A descriptive analysis of the study sample is given in (Table 2).

**Table 2:** Description of the study population

Variables	Values (N %)
Age* (Y) (N = 148)	41.7 ± 11.7
Sex N (%) (N = 148)	
Female	112 (75.7)
Male	36 (24.3)
Tooth type N (%) (N = 358)	
Molars	131 (36.6)
Premolars	129 (36)
Incisors	80 (22.3)
Canines	18 (5)
Coronal restoration type N (%) (N = 358)	
Filling inserted during plastic phase	250 (69.7)
Full coverage crown	85 (23.7)
Temporary or absent	22 (6.1)
Intracanal posts N (%) (N = 358)	58 (16.3)
Opposing tooth N (%) (N = 358)	
Present	318 (90.1)
Absent	34 (9.6)
Coronal restoration quality N (%) (N = 358)	
Adequate	159 (44.4)
Inadequate	199 (55.6)
Periapical status N (%) (N = 358)	
Healthy	100 (27.9)
Apical periodontitis	258 (72.1)

(N = 148): number of patients, (N = 358): number of teeth, *Y* years, *mean ± Standard deviation

A Total of 369 endodontically treated teeth were assessed. However, 11 teeth were excluded from the study because endodontic treatment has been achieved less than 1 year. Of the 358 endodontically treated teeth included in the study, Molars were predominant 131 (36.6%).

AP was detected on 258 (72.1%) of the studied endodontically treated teeth. Coronal restoration with open margins and recurrent decay were recorded in 68 (19%) and 45 (12.6%) of endodontically treated teeth respectively. 160 (65%) of the restored teeth matched CL II cavity design. (Table 3) shows descriptive data of coronal restoration and root canal filling quality.

**Table 3:** Descriptive data of coronal restoration and root canal filling quality in the study population

Variables	N (%) (N = 358)
Coronal restoration Index	
Adequate	159 (44.4)
Overhangs	32 (8.9)
Open margins	68 (19)
Non-anatomic	21 (5.9)
Recurrent decay	45 (12.6)
Fractured or missing restoration	27 (7.5)
Fractured tooth	6 (1.7)
Root canal filling quality	
Under and over-filling	218 (60.8)
Inadequate homogeneity	247 (69)
Inadequate conicity	251 (70.1)

Univariate regression analysis showed that inadequate coronal restoration, inadequate root canal filling quality, gingival inflammation and coronal restorations with CL II cavity design were identified as risk factors associated with higher rate of apical periodontitis. The results of unadjusted (univariable model) are shown in (Table 4).

**Table 4:** Univariate logistic Regression for the association of study variables with periapical status

Variable	Apical periodontitis N (%)	OR	95% CI	P value
Age (Years)		1.02	0.99–1.05	0.08
Sex				
Female	75 (66.4%)	1.11	0.50–2.44	0.78
Tooth type				
Incisors	57 (71.2%)	1		
Canines	11 (61.1%)	0.63	0.21–1.83	0.40
Premolars	94 (72.9%)	1.06	0.57–1.97	0.85
Molars	96 (73.3%)	1.10	0.59–2.05	0.74
Coronal restoration quality				
Adequate	105 (66%)	1		
Inadequate	153 (76.9%)	1.71	1.07–2.72	0.02
Coronal restoration type				
Temporary or absent	20 (90.9%)	1		
Filling inserted during plastic phase	169 (76.3%)	0.20	0.04–0.90	0.03
Full coverage crown	69 (81.2%)	0.43	0.09–2.03	0.28
Cavity design				
CL IV	1 (20%)	1		
CL I	20 (64.5%)	7.27	0.72–73.3	0.09
CL II	113 (70.6%)	9.61	1.07–88.3	0.04
CL III	31 (62%)	6.52	0.67–62.8	0.10
Gingival status				
Healthy	122 (66.3%)	1		
Gingival inflammation	126 (78.3%)	1.85	1.14–2.99	0.01
Periodontal status				
Healthy	207 (74.2%)	1		
Periodontal disease	38 (63.3%)	1.66	0.92–3.00	0.09
Intracanal posts				
Present	46 (79.3%)	1		
Absent	210 (70.7%)	0.63	0.31–1.24	0.18
Opposing tooth				
Present	225 (70.8%)	1		
Absent	28 (82.4%)	1.40	0.62–3.13	0.40
Root canal filling quality				
Under and over-filling	158 (78.6%)	2.57	1.66–4.002	< 0.001
Inadequate homogeneity	199 (80.6%)	3.65	2.24–5.95	< 0.001
Inadequate conicity	198 (78.9%)	2.92	1.79–4.76	< 0.001

Multivariate analysis (Table 5) with the dependent variable "apical periodontitis ", and coronal restoration quality, coronal restoration type, gingival status, CL II cavity design and root canal filling quality as explanatory variables, showed that teeth with inadequate coronal restoration, inadequate root canal filling length, inadequate root canal filling homogeneity, and with gingival inflammation had a (2.16) fold (95% CI 1.16–4.04, p = 0.01), (1.93) fold (95% CI 1.24–3.01, p = 0.004), (2.50) fold (95% CI 1.41–4.44, p = 0.002) and (2.05) fold (95% CI 1.08–3.91, p = 0.02) greater chance respectively of apical periodontitis than teeth with adequate coronal restoration, with adequate root canal filling quality and endodontically treated teeth with healthy gingival status.

**Table 5:** Multivariate logistic Regression for the association of the dependent variable "apical periodontitis" with a number of explanatory variables (coronal restoration quality, coronal restoration type, gingival status, CL II cavity design, root canal filling quality)

	Apical periodontitis (%)	OR	95% CI	P value
Coronal restoration quality				
Inadequate	153 (76.9%)	2.16	1.16–4.04	0.01
Coronal Restoration type				
Temporary or absent	20 (90.9%)	1		
Filling inserted during plastic phase	169 (76.3%)	0.36	0.06–1.97	0.24
Full coverage crown	69 (81.2%)	0.79	0.13–4.83	0.80
Gingival status				
Gingival inflammation	126 (78.3%)	2.05	1.08–3.91	0.02
Cavity design				
CL II	113 (70.6%)	0.82	0.49–1.35	0.44
Root canal filling quality				
Under and over-filling	218 (60.8%)	1.93	1.24–3.01	0.004
Inadequate homogeneity	247 (69%)	2.50	1.41–4.44	0.002
Inadequate conicity	251 (70.1%)	1.57	0.87–2.81	0.12

## Discussion

The current study aimed to investigate prevalence of AP and risk factors associated to AP in endodontically treated teeth in a Moroccan Adult subpopulation. The results of the present study indicate that (72.1%) of the root filled teeth had AP. In a Moroccan subpopulation, Chala et al. revealed that AP has been found in 63.79% of the root filled teeth, Jimenéz-Pinzón et al. found in a Spanish subpopulation AP in 65.8% of the endodontically treated [21, 32]. However, a lower AP prevalence was recorded in other epidemiological studies [3, 13]. When comparing the results of different epidemiological studies, some points should be considered: the use of different study sampling methodologies, variability of the evaluation criteria for apical periodontitis, different radiological examination techniques considered for the evaluation of periapical status, different practitioners endodontic training skills and different oral health awareness levels between populations. These features might explain the discrepancies observed between the results of different studies. In the present study, a high prevalence of AP was recorded, and this can be explained by an elevated number of cases with inadequate endodontic treatments and inadequate coronal restorations. Moreover, Endodontic treatments and coronal restorations were carried out by practitioners with different endodontic training qualifications, not all the treatments were undertaken in the University teaching hospital, many of them were provided in private practice or in state specialist hospitals.

**Fig. 1:**
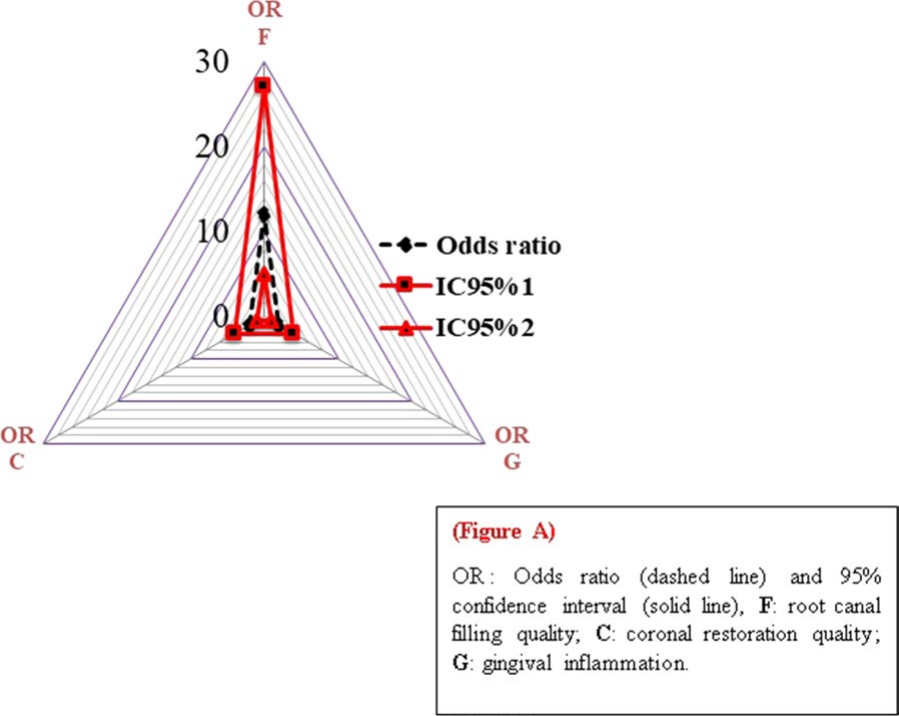
*OR* odds ratio (dashed line) and 95% confidence interval (solid line), *F* root canal filling quality, *C* coronal restoration quality, *G* gingival inflammation

As well as many studies, the present study revealed that both of the root canal filling and coronal restoration quality are equally important for the periapical health in endodontically treated teeth [9, 33]. According to the present study, when the root canal filling was inadequate, the good coronal seal did not prevent AP in endodontically treated teeth (Fig. 1).

El Merini et al. recorded in their study AP in 91. 5% of endodontically treated teeth with inadequate root canal treatment, and AP in their study was significantly associated with insufficient root canal fillings (P < 0.05) [5]. Moreover, in a Meta-analysis of previous cross-sectional studies published between 2012 and 2020, Jakovljevich et al., had shown a significant higher prevalence of AP in treated teeth with inadequate root canal treatment (OR = 4.65; 95% CI 2.75–7.84; p < 0.00001), and a significant higher prevalence of AP in ETT with poor coronal restoration (OR = 1.54; 95% CI 1.16–2.05, p = 0.003) [9], these findings are all in accordance with the results of the present study.

The interpretation of radiographs is the most common method used to evaluate AP in epidemiological studies [11, 34], clinical symptoms such as pain, swelling, sinus tract formation and tenderness are moderately specific. In the current study, periapical radiography was preferred because not only the presence of AP but also endodontic treatment and coronal restoration quality were assessed.

It’s conceivable that scoring the quality of coronal restoration from a radiograph is not possible with certainty as it only provides a two dimensional image, and this is supported by previous findings which stated a weak correlation between radiographic and clinical data related to coronal restoration quality [33, 35]. Therefore, AP and coronal restoration quality were assessed clinically and radiographically in the present study for a better accuracy. Furthermore, clinical and radiographic evaluation were both necessary for the assessment of the periodontal health status.

Association of AP and periodontal status was few investigated in endodontically treated teeth [15, 36]. In a study carried out by Virtanen et al., Periodontitis was among the significant explanatory factors for having apical periodontitis. The authors suggested that chronic inflammation has been persistent for a long time in the periodontal ligament causing a higher oral infection burden to patients, which might explain the connection found between AP and periodontitis [17].

The present study revealed that inadequate coronal restorations especially those with large proximal margins (CL II cavity design), and gingival inflammation increased the risk of AP in endodontically treated teeth. Costa et al. reported that gingival bleeding on probing was significantly associated to periapical status, however periodontal bone loss didn’t influence periapical health [18] and this is in accordance with the present study results. In similar context, Skupien et al. and Khalighinejad et al. confirmed in two recent studies the high predictable survival rate of endodontically treated teeth after root canal treatment in cases with a healthy periodontium [15, 36].

From the above findings, we can suggest that gingival inflammation may be associated with progressing caries and bacterial biofilm activity on approximal surfaces of teeth, this is in agreement with the fact that gingival wall in proximal restorations with overhangs is the most common site of plaque accumulation and the development of recurrent caries as reported by Mjör [37, 38]. We can also suggest, that in CLII cavity design, the coronal structure loss is relatively high, which may contribute to a more severe coronal leakage in the years following endodontic treatment achievement. This concurs with Al Nuaimi et al. findings, in their study teeth with less than 30% of their original tooth volume had a 2.58-fold (95% CI 1.026–6.487) greater chance of apical periodontitis than those that had more than 30% of volume at the 1-year follow up period.

In endodontically treated teeth several factors can interact with each other, absence of immune defense mechanisms related to vital pulp tissue, coronal restoration with inadequate proximal margins, gingival disease, and inadequate root canal filling if concurrent can potentiate diffusion of micro-organisms in relation to gingival inflammation into the root canal space. Gingivitis and apical periodontitis are both a bacterial biofilm-induced diseases, bacteria can damage host cells and/or the intercellular matrix of connective tissue through their pathogenic mechanisms; chemical mediators involved in the induction of bone resorption can also be released in response to the stimulation by bacterial components [39, 40].

In the same context, it was stated that root canals are invaded by oral microorganisms after pulpal exposure [40]. The colonizing species can grow and make adhesions to the root canal walls, detachment of microorganisms occurs progressively and lead to spread of infection toward the apex [41]. These inflammatory changes occurring in the periapical periodontium are responsible for the disease.

The host immune defenses can deal with infection spread from the infected root canal. However, Microbial species remaining beyond the reaches of an active microcirculation within the root canal space, can not be eradicated [42]. According to the present study findings, we can suggest that coronal restorations with inadequate proximal margins especially when gingival inflammation is concurrent and the root canal filling is inadequate are the most affected by bacterial leakage, therefore endodontic treatment failure. Thus the importance of a well sealing coronal restorations for lasting success of endodontic treatment that was stressed by several reports was evident in this study as well [43, 44].

Recontamination of the root canal system by coronal leakage has been emphasized. Root canal filling leakage studies reported root canal system reinfection due to micro-organisms and their products after root canal filling exposure [45, 46]. Although there is a need to establish the relationship between bacterial leakage and periapical inflammation, thereby the combination of our results and those of other studies [47, 48] provide a clinical significance to the in vitro findings.

AP impact on the general health has been widely investigated, it has been reported that periapical chronic inflammation could induce or perpetuate an elevated chronic systemic inflammatory status, contributing to increased insulin resistance and poor glycaemic control in Diabetic patients [49, 50]. Endodontic infections were also recorded to be statistically associated with cardiovascular diseases [17]. Therefore, there is a persistent need for resolving this oral health condition since there is an increasing evidence of the association between AP and impaired systemic health [51, 52].

Frisk reported that association between the type of coronal restoration and AP in endodontically treated teeth has significance for recurrent caries [53]. However, contrarily to previous studies findings, the type of coronal restoration in the present study was not associated with AP [54].

Root canal filling > 2 mm short from the radiographic apex and root canal fillings with inadequate homogeneity were associated with AP in the current study, these results agree with the findings of previous studies [6] supporting the necessity of instrumentation closer to the radiographic apex and adequate filling of the root canal system. Histopathologic observation studies reported in cases of under-fillings, bacterial proliferation in unsealed spaces of the root canal system, over-fillings are also associated with extrusion of infected debris toward periapical tissues [1, 55]. Eventhough, AP healing can be achieved even when bacteria are remaining in the root canal, and this can be explained by the fact that residual microorganisms involved in the root canal infection are not able to exert their pathogenic mechanisms when the root canal filling is carried out [56], because a tridimensional and homogenous root canal filling can entomb bacteria in the canal denying them access to periradicular tissues [57].

In the literature, a few reports had been published on the influence of the root canal filling conicity on periapical health [58, 59], and controversial results were reported in this respect. In the present study, inadequate root canal filling conicity was associated to apical periodontitis when studied as a separate variable, in combination with the above findings we could suggest that root canal filling conicity can affect periapical health when root canal filling length and homogeneity are inadequate.

## Conclusions

Root canal filling and coronal restoration adequacy, gingival inflammation are the most risk factors of apical periodontitis in endodontically treated teeth according to the present study. In addition to many surveys reportings, the present study gives an overview of the high prevalence of apical periodontitis still recorded despite restorative and endodontic advances. High prevalence of apical periodontitis within endodontically treated teeth in the current study, should make practitioners aware of the multifactorial aspect of the disease, therefore key features in relation to restorative and endodontic procedures must be well managed to improve periapical health conditions.

## Data Availability

The datasets used for the current study are available from the corresponding author on reasonable request.

## Abbreviations

AP: Apical periodontitis
ETT: Endodontically treated teeth
